# Malvidin attenuates trauma‐induced heterotopic ossification of tendon in rats by targeting Rheb for degradation via the ubiquitin‐proteasome pathway

**DOI:** 10.1111/jcmm.18349

**Published:** 2024-04-30

**Authors:** Huaji Jiang, Yan Ding, Xuemei Lin, Qinyu Tian, Yakui Liu, Hebei He, Yongfu Wu, Xinggui Tian, Stefan Zwingenberger

**Affiliations:** ^1^ Yue Bei People's Hospital Postdoctoral Innovation Practice Base Southern Medical University Guangzhou China; ^2^ Department of Diagnostics, School of Medicine Hunan University of Medicine Huaihua Hunan Province China; ^3^ Department of Pediatric Orthopedics Guangzhou Women and Children's Medical Center, Guangzhou Medical University Guangzhou China; ^4^ Department of Orthopaedics and Traumatology, Faculty of Medicine The Chinese University of Hong Kong Hong Kong SAR China; ^5^ Center for Translational Bone, Joint and Soft Tissue Research, University Hospital Carl Gustav Carus at Technische Universität Dresden Dresden Germany; ^6^ Department of Sports Medicine, The First Affiliated Hospital, Guangdong Provincial Key Laboratory of Speed Capability, The Guangzhou Key Laboratory of Precision Orthopedics and Regenerative Medicine Jinan University Guangzhou PR China; ^7^ University Center of Orthopaedic, Trauma and Plastic Surgery, University Hospital Carl Gustav Carus at Technische Universität Dresden Dresden Germany

**Keywords:** heterotopic ossification, mTORC1, proteasome, tendon, ubiquitination

## Abstract

The pathogenesis of trauma‐induced heterotopic ossification (HO) in the tendon remains unclear, posing a challenging hurdle in treatment. Recognizing inflammation as the root cause of HO, anti‐inflammatory agents hold promise for its management. Malvidin (MA), possessing anti‐inflammatory properties, emerges as a potential agent to impede HO progression. This study aimed to investigate the effect of MA in treating trauma‐induced HO and unravel its underlying mechanisms. Herein, the effectiveness of MA in preventing HO formation was assessed through local injection in a rat model. The potential mechanism underlying MA's treatment was investigated in the tendon‐resident progenitor cells of tendon‐derived stem cells (TDSCs), exploring its pathway in HO formation. The findings demonstrated that MA effectively hindered the osteogenic differentiation of TDSCs by inhibiting the mTORC1 signalling pathway, consequently impeding the progression of trauma‐induced HO of Achilles tendon in rats. Specifically, MA facilitated the degradation of Rheb through the K48‐linked ubiquitination‐proteasome pathway by modulating USP4 and intercepted the interaction between Rheb and the mTORC1 complex, thus inhibiting the mTORC1 signalling pathway. Hence, MA presents itself as a promising candidate for treating trauma‐induced HO in the Achilles tendon, acting by targeting Rheb for degradation through the ubiquitin‐proteasome pathway.

## 1 INTRODUCTION

Heterotopic ossification (HO) is a complex pathological process defined as the formation of extraskeletal bone in muscles and soft tissues.^1^, ^2^ HO is generally categorized into two types: traumatic and genetic.^2^ Trauma‐induced HO, the most prevalent but mechanistically least understood, typically occurs following events such as severe burns, surgery, fractures or joint replacements.^2^, ^3^ Injury or rupture of tendons is a prevalent musculoskeletal disorder, and the repair process is typically characterized by a slow rate of healing.^3^ HO of tendons is a frequent and severe complication following tendon injury, hindering normal tendon repair and significantly impacting tendon function.^4^ Conservative treatments like physical therapy and medication often alleviate tendon ossification but do not provide complete elimination.^4^ The current approach to HO involves predominantly surgical removal of ectopic bone tissue, causing additional trauma and increasing the risk of HO recurrence.^4^ Consequently, there is an urgent need to develop and implement effective and reliable pharmacological preventive measures based on the pathogenesis of HO. Histologically, trauma‐induced HO is thought to advance through four stages: inflammation, chondrogenesis, osteogenesis and maturation, akin to the process of fracture repair.^3^ Focusing treatment on the initial stages of HO development might yield more favourable outcomes than attempting to eliminate already formed bone tissue.^5^ In clinical practice, nonsteroidal anti‐inflammatory drugs (NSAIDs) like celecoxib and indomethacin have been extensively employed to prevent traumatic HO.^2^, ^5^ Nevertheless, their effectiveness is limited, and the accompanying risk of complications is considerable.^2^, ^5^ Additionally, when employed in the treatment of HO, NSAIDs may negatively impact the healing process of fractures.^2^ Hence, it is imperative to develop specific non‐NSAIDs anti‐inflammatory drugs that target the initial inflammatory stage for attenuating or preventing trauma‐induced HO of tendons.

Previous studies have shown that a variety of cells participate in HO and various signalling pathways have also been reported.^2^ In the trauma‐induced HO model of Achilles tendon, tendon‐derived stem cells (TDSCs) are identified as the principal progenitor cells, and their osteogenic differentiation assumes a critical role in the development of tendon HO.^4^, ^6^ Our previous investigation demonstrated that inhibiting the mammalian target of rapamycin complex 1 (mTORC1) signalling in TDSCs can prevent the development of Achilles tendon HO, presenting it as a promising therapeutic target.^7^ Furthermore, mammalian target of rapamycin (mTOR) plays a role in the initial inflammatory stage of HO formation.^8^ Building on this understanding, targeting the mTORC1 signalling pathway of TDSCs appears to be a potential strategy for mitigating the inflammatory response and treating HO of Achilles tendon. Malvidin (MA) is a common *O‐methylated anthocyanin* with antioxidant and anti‐inflammatory properties.^9^, ^10^, ^11^ Recently, several studies have suggested that MA may exhibit therapeutic effects in various inflammatory diseases, such as peptic ulcer,^12^ osteoarthritis^13^ and acute liver injury.^14^ Hence, MA appears to hold therapeutic potential for HO by inhibiting the initial inflammatory stage. However, the question of whether it can mitigate or prevent trauma‐induced HO of Achilles tendon by targeting potential signalling pathways of TDSCs necessitates further exploration. This study aimed to assess the effect of MA in the treatment of trauma‐induced HO of Achilles tendon and to elucidate its possible underlying mechanisms.

This study hypothesized that MA could target resident TDSCs via inhibiting the mTORC1 signalling pathway to suppress the progression of trauma‐induced HO of Achilles tendon.

## 2 RESULTS

### 2.1 MA inhibits trauma‐induced HO formation of Achilles tendon in rats

**FIGURE 1:**
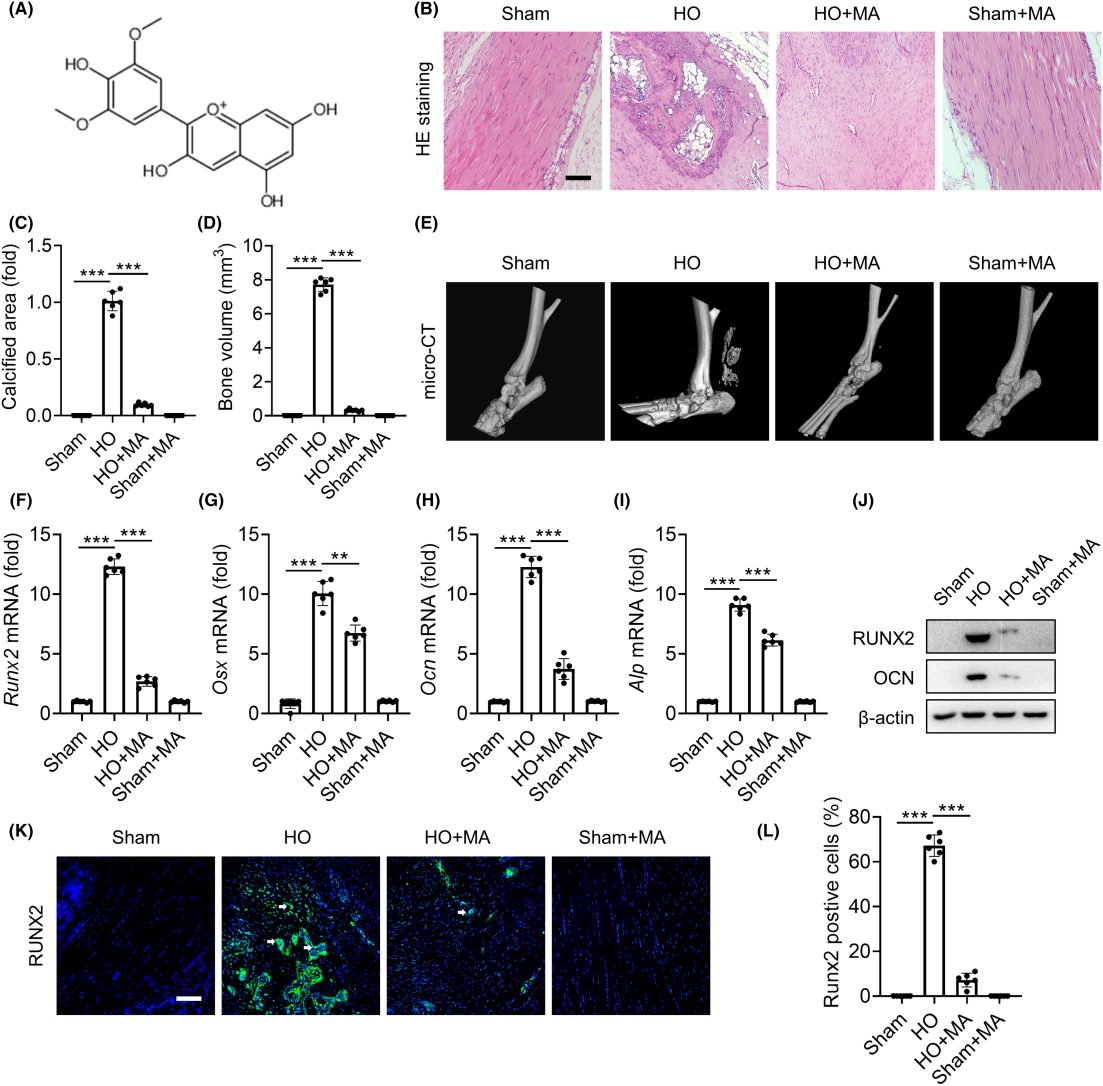
Malvidin (MA) inhibits trauma‐induced heterotopic ossification (HO) formation of Achilles tendon in rats after 10 weeks of treatment. (A) The chemical structural formula of MA. (B, C) HE staining images were utilized to assess the HO formation of Achilles tendon and the corresponding quantification of calcified area based on staining images. (D, E) The bone volume of HO formation at Achilles tendon area and the representative μCT 3D reconstruction images to evaluate the effect of MA on the HO formation. (F–I) The effect of MA on the expression of osteogenic differentiation‐related genes (*Runx2*, *Osx*, *Ocn* and *Alp*) of Achilles tendon tissue was detected by qRT‐PCR. Expression levels were normalized to those of the GAPDH housekeeping gene, and the data were analysed using the ΔΔ‐Ct method.^15^ (J) The effect of MA on osteogenic differentiation‐related proteins (RUNX2 and OCN) of Achilles tendon tissue was assessed by WB. (K, L) Immunofluorescence images of pathological sections for RUNX2 and corresponding quantification results were examined to evaluate the osteogenic expression influenced by MA in vivo; the white arrows indicated the RUNX2‐positive cells. Data are expressed as the means ± SD. ** *p* < 0.01, *** *p* < 0.001. Scale bar = 100 μm.

The chemical structure of MA is shown in Figure 1A. After 10 weeks of treatment, HE staining showed that the Sham+MA group exhibited characteristics consistent with the Sham group, displaying healthy Achilles tendon tissue, characterized by a parallel longitudinal arrangement of collagen fibres and a uniform distribution of tenocytes along the collagen fibres' long axis. Conversely, the trauma‐induced HO group displayed disordered collagen bundles, fragmented collagen fibres and significant calcified bone tissue. In the HO+MA group, new bone tissue was also observed within the collagen fibres of the Achilles tendon, with significantly reduced calcification area and degree compared to the untreated HO group (Figure 1B,C). μCT 3D reconstruction images showed that substantial bone tissue formation in the Achilles tendon of the HO group, while only a limited amount of bone tissue formed in the HO+MA group. No bone tissue was observed in the Achilles tendon area of the Sham and the Sham+MA groups. μCT quantitative analysis of the Achilles tendon region, defined as the region of interest (ROI) in this study, indicated that the HO+MA group was significantly higher than the Sham and Sham+MA groups, but significantly lower than the HO group (Figure 1D,E). Quantitative reverse transcription PCR (qRT‐PCR) results of osteogenic differentiation‐related genes *Runx2*, *Osx*, *Ocn and Alp* (Figure 1F–I) and the Western blotting (WB) results of osteogenic differentiation‐related proteins RUNX2 and OCN (Figure 1J) both showed that MA significantly suppressed high osteogenic differentiation in the trauma‐induced HO formation of Achilles tendon. The immunofluorescence of pathological sections for RUNX2 confirmed that MA also significantly reduced osteogenic expression in the HO+MA group compared with the HO group in vivo (Figure 1K,L). Based on these results, it can be concluded that the rat trauma‐induced HO model of Achilles tendon was successfully established, and MA effectively inhibited the trauma‐induced HO formation of Achilles tendon in this study.

### 2.2 MA inhibits IL‐1β‐mediated osteogenic differentiation of TDSCs

**FIGURE 2:**
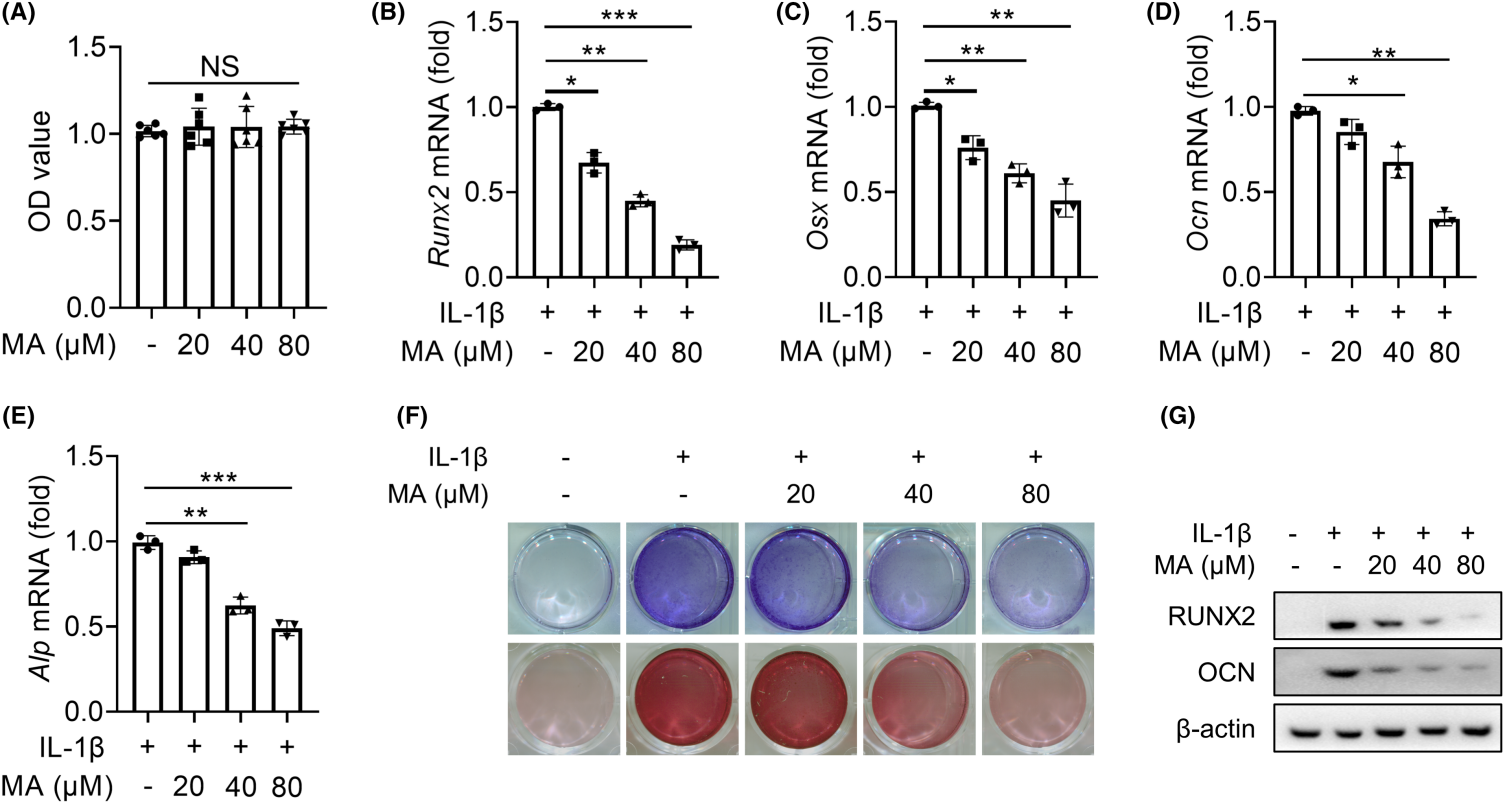
Malvidin (MA) inhibits IL‐1β‐mediated osteogenic differentiation of tendon‐derived stem cells (TDSCs) in vitro. (A) The CCK‐8 experiment was utilized to assess the effect of MA on the proliferation of TDSCs by measuring the optical density (OD) value to establish the safety concentration of MA. (B–E) The effect of MA on IL‐1β‐mediated osteogenic differentiation genes (Runx2, Osx, Ocn and Alp) expression of TDSCs was assessed by qRT‐PCR, respectively. (F) ALP staining and Alizarin red staining were utilized to assess the effect of MA on the IL‐1β‐mediated osteogenic differentiation of TDSCs. (G) The effect of MA on IL‐1β‐mediated osteogenic differentiation of TDSCs was assessed by WB. * *p* < 0.05, ** *p* < 0.01, *** *p* < 0.001.

To reveal the molecular mechanism of MA alleviating the progression of trauma‐induced HO of Achilles tendon, the resident progenitor cells of tendon‐derived stem cells (TDSCs) were selected as target cells for in vitro experiments. CCK‐8 experiment established a safe concentration range for MA (0–80 μM) which had no significant inhibitory effect on the proliferation of TDSCs (Figure 2A). IL‐1β plays a crucial role as a pro‐inflammatory factor in the development of trauma‐induced HO,^2^ and can promote the osteogenic differentiation of TDSC in vitro.^16^ Therefore, it was selected to stimulate TDSCs to simulate their inflammatory osteogenic pathological microenvironment in trauma‐induced HO of Achilles tendon in this study. qRT‐PCR results showed that MA significantly decreased the expression of IL‐1β‐mediated osteogenic differentiation factors of *Runx2*, *Osx*, *Ocn and Alp* genes in a dose‐dependent manner (Figure 2B–E). ALP and alizarin red staining showed that MA decreased the expression of osteogenic differentiation factor ALP and calcium salt deposition mediated by IL‐1β in a dose‐dependent manner (Figure 2F). WB results also showed that MA significantly decreased the protein expression of RUNX2 and OCN (Figure 2G). These findings collectively reinforced that MA inhibited the osteogenic differentiation of TDSCs in a dose‐dependent manner in vitro.

### 2.3 MA inhibits the mTORC1 signalling pathway by promoting Rheb proteasomal degradation

**FIGURE 3:**
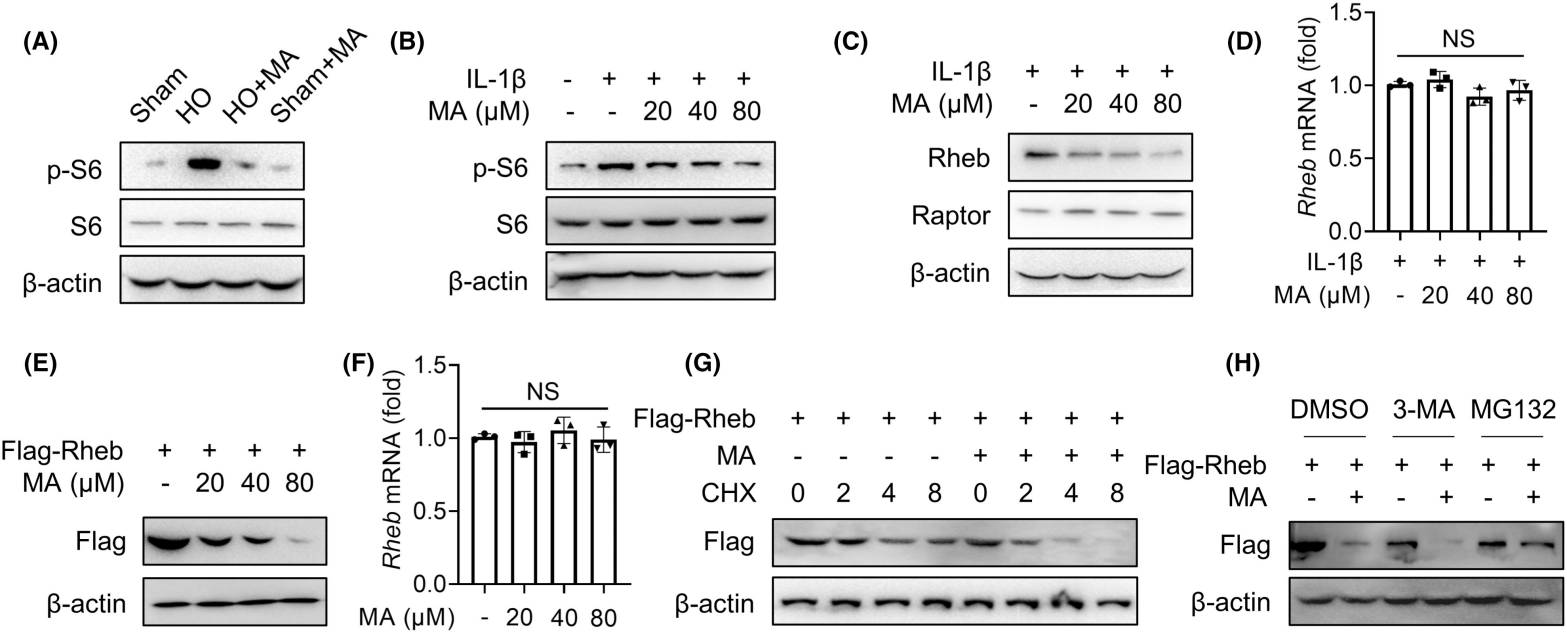
Malvidin (MA) inhibits the mTORC1 signalling pathway by promoting Rheb proteasomal degradation. (A) The effect of MA on the downstream protein of the mTORC1 pathway S6 and its phosphorylated form p‐S6 of Achilles tendon tissue was assessed by WB. (B) Western blotting (WB) was employed to assess the impact of MA on mTORC1 downstream proteins S6 and p‐S6 in TDSCs in vitro. (C) The effect of MA on the subunit Raptor and upstream Rheb protein of mTORC1 in TDSCs were examined by WB. (D) qRT‐PCR was utilized to detect the impact of MA on the Rheb gene expression in TDSCs. (E, F) The Flag‐Rheb plasmid was transfected into HEK293T cells; subsequently, the effect of MA on (E) exogenous Rheb protein and (F) the transcript level of Rheb mRNA was evaluated by WB and qRT‐PCR, respectively. (G) The effect of MA on exogenous Rheb protein in HEK293T cells transfected with Flag‐Rheb plasmid was evaluated by WB, under the action of protein synthesis inhibitor cycloheximide (CHX) to prevent new protein synthesis. (H) HEK293T cells were transfected with Flag‐Rheb plasmid and then treated with MA and autophagy inhibitor 3‐MA or proteasome inhibitor MG132, respectively, to investigate the degradation pathway of Rheb protein by MA. Data are expressed as the means ± SD. ‘NS’ means no significance.

Our previous research has shown that the inhibition of mTORC1 signalling in TDSCs attenuates trauma‐induced HO formation of Achilles tendon.^7^ To investigate whether MA inhibits HO formation by regulating the mTORC1 signalling pathway of TDSCs in this study, the effect of MA on the the downstream protein of the mTORC1 pathway S6 and its phosphorylated form, p‐S6, which is activated by mTORC1,^17^ was detected by WB. The results showed that MA inhibited the expression of p‐S6 in vivo, indicating MA inhibits mTORC1 activation in vivo (Figure 3A). In vitro experiments also showed that MA dose‐dependently inhibited the expression of p‐S6 in TDSCs (Figure 3B). These findings indicated that MA inhibited the mTORC1 signalling pathway both in vivo and in vitro. To pinpoint the special target acting on the mTORC1 signalling pathway, the effects of MA on its subunit Raptor and upstream Rheb protein were examined, respectively.^18^ The results indicated that MA acted by inhibiting the upstream Rheb protein rather than the complex subunit Raptor^18^ (Figure 3C). However, qRT‐PCR results showed that MA had no obvious inhibitory effect on the transcription expression of *Rheb* gene mediated by IL‐1β in vitro (Figure 3D). To investigate the molecular mechanism of Rheb protein degradation by MA, HEK293T cells were utilized due to their ability to be transfected with target proteins in specific plasmid vectors.^19^ Further investigation showed that MA had a concentration‐dependent degradation of transfected exogenous Rheb protein in HEK293T cells (Figure 3E), but had no significant effect on the transcript level of Rheb mRNA (Figure 3F). Under the action of protein synthesis inhibitor cycloheximide (CHX) to prevent new protein synthesis, MA still suppressed the protein expression of Rheb in a time‐dependent manner in HEK293T cells (Figure 3G). These findings suggested that MA exerted its therapeutic effect by degrading Rheb protein. Since there are two main systems of protein degradation in eukaryotes (the proteasome and autophagy lysosomal pathways), we next determined in which pathway MA induces the degradation of Rheb protein. The effect of MA‐mediated Rheb degradation was completely inhibited by the proteasome inhibitor MG132, but not by autophagy inhibitor 3‐methyladenine (3‐MA) (Figure 3H). These findings indicated that MA blocked the mTORC1 signalling pathway by promoting the proteasomal degradation of Rheb protein.

### 2.4 MA mediates proteasomal degradation of Rheb protein by inducing K48 ubiquitination

Ubiquitination is a prerequisite for mediating proteasome degradation. Subsequently, we explored whether MA promotes Rheb protein proteasome pathway degradation by affecting its ubiquitination.

**FIGURE 4:**
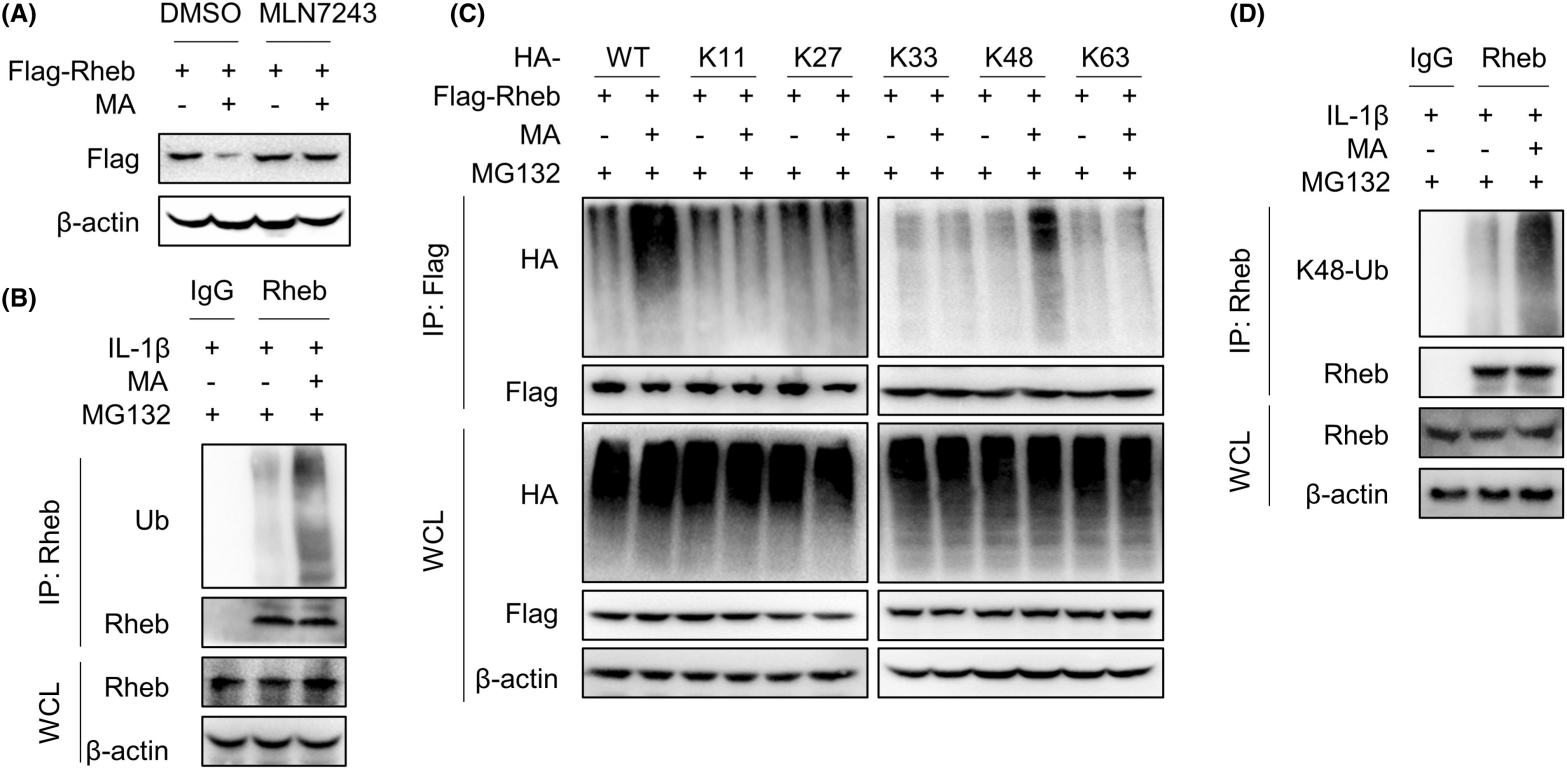
Malvidin (MA) mediates proteasomal degradation of Rheb protein by inducing its K48‐linked ubiquitination. (A) Investigating MA‐mediated degradation of exogenous Rheb protein transfected in HEK293T cells by inhibiting ubiquitin‐activating enzyme activity using its inhibitor MLN7243. (B) Under inhibition of proteasome activity by MG132, immunoprecipitation (IP) experiments were performed to validate whether MA could mediate the interaction between IL‐1β‐induced endogenous Rheb protein and ubiquitin (Ub) in TDSCs. (C) Flag‐Rheb and HA‐tagged ubiquitinated plasmids (WT, K11, K27, K33, K48, K63) were transfected into HEK293T cells to investigate the specific ubiquitin involved in MA‐mediated Rheb degradation by IP experiment, demonstrating that MA facilitates K48 ubiquitination of exogenous Rheb protein. (D) In the presence of MG132 inhibiting proteasome activity, an IP experiment was performed to validate the effect of MA on the interaction between IL‐1β‐mediated endogenous Rheb and K48 ubiquitin in TDSCs.

WB experiment results showed that MA‐mediated degradation of Rheb could be blocked by the ubiquitin inhibitor MLN7243 in HEK293T cells, indicating that MA promotes the degradation of Rheb via the ubiquitin‐proteasome pathway (Figure 4A). Subsequently, the identified mechanism was further validated in the primary resident progenitor cells of TDSCs. The investigated mechanism was confirmed in TDSCs through immunoprecipitation (IP) experiments, wherein MA effectively increased the ubiquitination of endogenous Rheb protein mediated by IL‐1β (Figure 4B). Further exploration showed that MA specifically increased the K48‐linked polyubiquitination of exogenous Rheb protein in HEK293T cells (Figure 4C). Meanwhile, IL‐1β activated the proteasome system in TDSCs (Figure S1). This finding was also validated in TDSCs, wherein MA enhanced the K48‐linked polyubiquitination of endogenous Rheb mediated by IL‐1β (Figure 4D). These findings suggest that MA promotes K48‐linked polyubiquitination of Rheb, thereby facilitating its degradation via the proteasome pathway.

### 2.5 MA promotes the K48‐linked ubiquitination of Rheb by regulating deubiquitinase USP4

**FIGURE 5:**
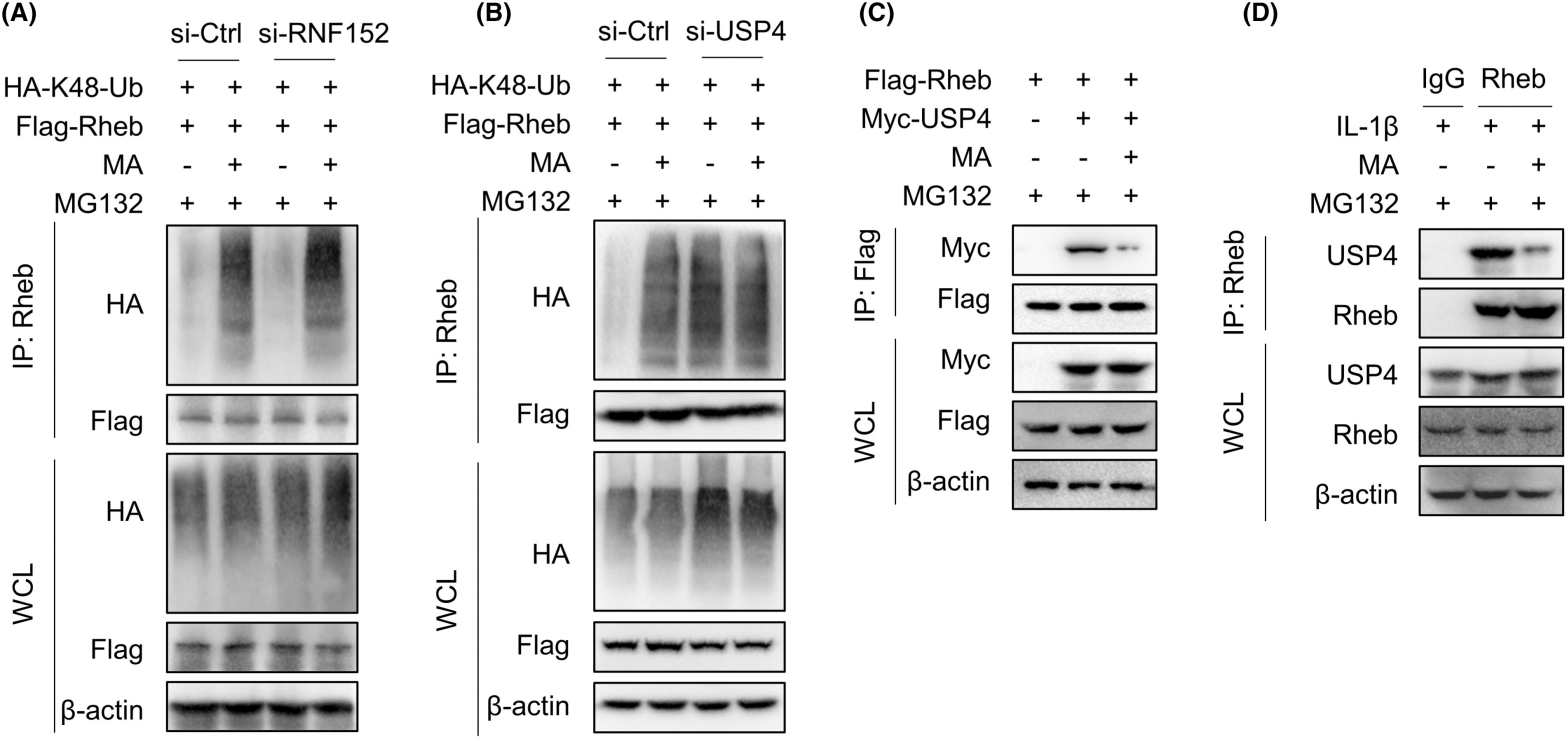
MA promotes the K48‐linked ubiquitination of Rheb by regulating deubiquitinase USP4. (A, B), E3 ubiquitin ligase RNF152 (A) and deubiquitinase USP4 (B) were silenced, respectively, to investigate the mechanism of MA regulating K48 ubiquitination of Rheb. (C) Flag‐Rheb and Myc‐USP4 plasmids were transfected into HEK293T cells, and the effect of MA on the interaction between Rheb and USP4 was investigated by IP experiments. (D) The effect of MA on the interaction of USP4 and Rheb in TDSCs was further validated by IP experiment in the presence of MG132.

The ubiquitination of Rheb protein is reported to be regulated by the E3 ubiquitin ligase RNF152 and the deubiquitinase USP4.^20^, ^21^ To explore whether MA regulates ubiquitination and proteasomal degradation of Rheb via RNF152 and/or USP4, RNF152 and USP4 were, respectively, silenced by corresponding siRNA to examine the interaction between Rheb protein and K48 ubiquitin in HEK293T cells. The results showed that MA continued to promote the K48‐linked ubiquitination of Rheb even when E3 ubiquitin ligase RNF152 was silenced (Figure 5A). However, when USP4 was silenced, MA failed to reverse the K48‐linked ubiquitination of Rheb (Figure 5B). These results suggest that MA regulates K48‐linked ubiquitination of Rheb by regulating USP4, rather than RNF152. Further investigation found that MA significantly inhibited the interaction between Rheb and USP4 in HEK293T cells (Figure 5C). This finding was further validated in TDSCs, wherein MA significantly inhibited the interaction between USP4 and Rheb mediated by IL‐1β, in the presence of MG132 (Figure 5D). Together, MA promotes the K48‐linked ubiquitination of Rheb by regulating deubiquitinase USP4 in TDSCs.

### 2.6 MA inhibits the mTORC1 signalling pathway by intercepting the interaction of Rheb and mTOR

**FIGURE 6:**
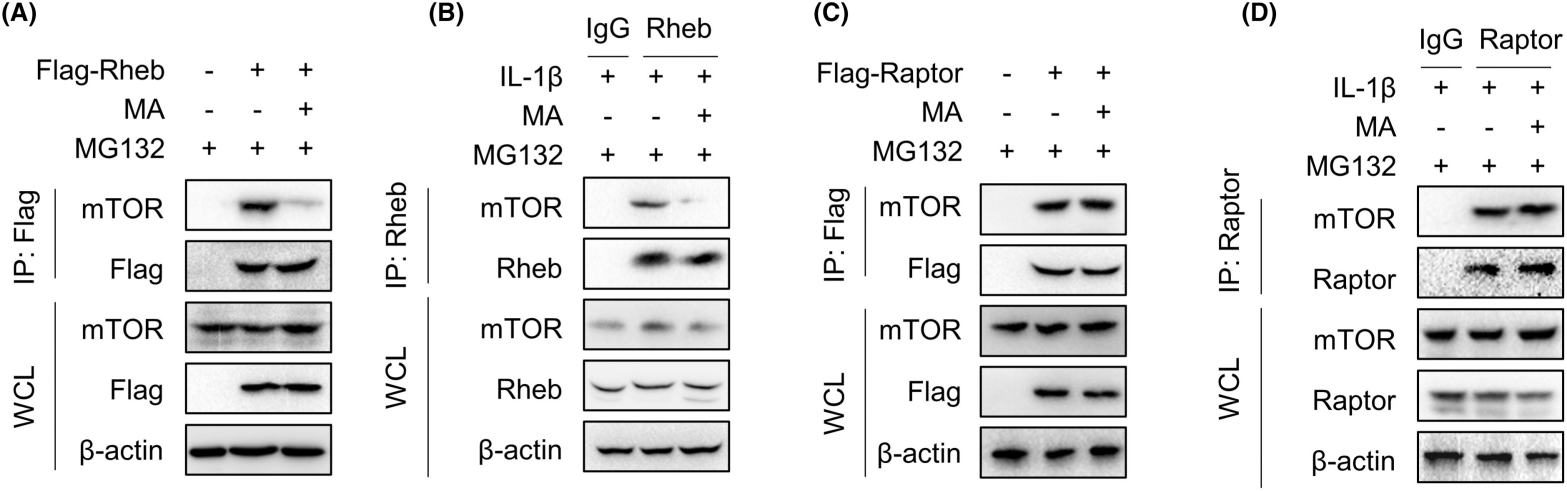
MA inhibits the mTORC1 signalling pathway by intercepting the interaction of Rheb and mTOR. (A) IP experiments investigated the effect of MA on the interaction between exogenous Rheb protein and mTOR transfected into HEK293T cells. (B) This above effect was further validated in TDSCs where the interaction between endogenous Rheb protein and mTOR induced by IL‐1β was assessed. (C) IP experiments investigated the effect of MA on the interaction between exogenous Raptor protein and mTOR transfected into HEK293T cells, (D) and the impact was also validated in TDSCs between IL‐1β‐mediated endogenous Raptor protein and mTOR.

IP experiments were performed to investigate whether MA, aside from promoting the degradation of Rheb protein, affects the interaction between Rheb and mTOR to inhibit the mTORC1 signalling pathway. In the presence of MG132, which inhibited proteasome activity to block the protein degradation pathway, MA markedly suppressed the interaction between mTOR and exogenous Rheb in HEK293T cells (Figure 6A). This effect was validated in TDSCs, where MA significantly inhibited the interaction between endogenous Rheb and mTOR induced by IL‐1β (Figure 6B). In contrast, MA failed to inhibit the interaction between exogenous Raptor and mTOR in HEK293T cells (Figure 6C). This effect was corroborated in TDSCs, where MA failed to inhibit the interlinkage between Raptor and mTOR (Figure 6D). These results indicate that MA inhibits the mTORC1 signalling pathway by intercepting the interaction of Rheb and mTOR.

## 3 DISCUSSION

In the present study, the rat trauma‐induced HO of Achilles tendon model was successfully established by the percutaneous Achilles tendon puncture method. MA demonstrated inhibitory effects on the formation of trauma‐induced HO in the Achilles tendon of rats. Mechanistically, MA hindered osteogenic differentiation of TDSCs, and consequently inhibited HO formation by suppressing the mTORC1 signalling pathway. Further investigations revealed that MA facilitated the K48‐linked ubiquitination of Rheb by modulating the deubiquitinase USP4, resulting in the degradation of Rheb through the proteasome pathway. Additionally, MA directly intercepted the interaction between Rheb and the mTORC1 complex. These findings suggest that MA holds potential as a therapeutic agent for trauma‐induced HO by targeting Rheb for degradation via the ubiquitin‐proteasome pathway.

Tendon is a compositionally complex tissue whose essential role is to perform the mechanical function, that is, by transferring force from the muscle to the bone, thereby converting muscle contraction into joint motion.^22^ The mature healthy tendon is mainly composed of an organized extracellular matrix, 95% of which are type I collagen fibres, which endow the tissue with high strength and good flexibility and elasticity.^23^, ^24^ The occurrence of HO in the tendon significantly impacts its mechanical strength, flexibility and elasticity. In cases of trauma‐related tendon ossification, it often results in tendon rupture, restricted mobility, chronic pain, compromised tendon function and other clinical symptoms.^4^ Although its pathogenesis has not been elucidated, the aetiology of HO of tendon is generally considered to be a failed tissue repair process, similar to fracture repair, involving trauma/injury, inflammation, stem cell recruitment, cartilage differentiation and ossification formation.^25^ Hence, developing effective interventions during the early stages of trauma‐induced HO initiation is crucial, which might yield more favourable outcomes than attempting to eliminate already formed bone tissue.^26^ Once bone tissue has formed, reversing the process becomes challenging, and surgical removal becomes the only option, leading to additional trauma and an increased risk of recurrence.^1^, ^25^, ^26^

In this study, the trauma‐induced HO model was established using percutaneous Achilles tendon puncture.^27^ Previous studies have shown that the incidence of HO is higher in males compared to females, possibly attributed to variations in muscle mass, physical activity levels and hormone levels affecting the signalling pathways of osteogenesis.^2^ Hence, only male rats were selected to enhance potential clinical relevance and mitigate potential effects associated with hormone levels. At the observation time point, substantial bone tissue formation was observed in the Achilles tendon of the puncture‐only (HO) group, affirming that the trauma‐induced HO model was successfully established. Inflammation is an important microenvironmental change during the development of HO.^2^ Trauma leads to local and systemic inflammatory states, leading to an increase in inflammatory cytokines, such as IL‐1β, which may lead to abnormal activation of soft tissue mesenchymal stem cells into the osteogenic lineage, thereby initiating the occurrence of ectopic ossification. IL‐1β is a pleiotropic pro‐inflammatory cytokine that can stimulate osteogenic differentiation of MSCs in vitro.^28^ More importantly, an in vitro study has also shown that IL‐1β can promote the osteogenic differentiation of Achilles tendon‐resident progenitor cells TDSCs.^16^ Therefore, IL‐1β was chosen to simulate the pathological process of inflammatory osteogenesis of TDSCs in the HO of Achilles tendon. When TDSCs were treated with IL‐1β alone, the expression of osteogenic differentiation significantly increased, indicating that it can simulate the microenvironment of inflammation‐induced osteogenic differentiation and that inflammatory factors can promote HO formation in the Achilles tendon. The rationale behind employing NSAIDs to prevent trauma‐induced HO is based on the theory that mitigating inflammation will correspondingly reduce HO formation.^1^ MA demonstrates anti‐inflammatory effects, suggesting its potential for the treatment of trauma‐induced HO.

In mouse and rat models of tendon‐related research, commonly employed administration methods include intraperitoneal injection,^27^ oral administration^29^ and local injection.^30^, ^31^ Moreover, our previous studies have indicated that the safety dosage for local injection therapy is often aligned within the safety dosage established in vitro cell experiments.^30^, ^31^ This approach helps prevent local toxic reactions caused by overdose and minimizes systemic side effects due to its high bioavailability. Therefore, local injection administration of MA was employed and employing the maximum safe dose established in in vitro experiments for in vivo experiments. The Sham+MA group exhibited characteristics of healthy Achilles tendon tissue and low expression of osteogenic differentiation, similar to the Sham group, indicating the non‐toxic nature of MA to Achilles tendon via local injection administration. However, MA demonstrated a significant inhibitory effect on trauma‐induced HO formation and the elevated expression of osteogenic genes in the HO+MA compared with the HO group. Consequently, MA emerges as a safe therapeutic agent for hindering trauma‐induced HO formation in the Achilles tendon of rats via local injection administration.

HO formation does not result from the precipitation of inorganic ions but from active cell‐mediated processes in which resident progenitor cells with multilineage differentiation potential may play a decisive role.^2^, ^32^ Tendon is a low‐cellularized tissue containing predominantly tenocytes and progenitor cell populations, in which tenocytes are major contributors to extracellular matrix remodelling in tendon.^24^ The resident progenitor cells of TDSCs represent precursors of tenocytes, possessing self‐renewal capabilities and the potential for multi‐differentiation in tendon formation, osteogenesis and chondrogenic differentiation.^4^, ^24^, ^25^ The osteogenic differentiation of TDSCs has been identified as crucial in HO formation of tendon.^4^, ^24^, ^25^ Moreover, our previous studies have revealed that the significant role of osteogenic differentiation of TDSCs in both the treatment of tendon‐bone healing disease^33^ and the pathogenesis of HO in the Achilles tendon.^7^ In this study, the in vitro experiments showed that MA inhibited the osteogenic differentiation of TDSCs. This finding holds significant implications for the treatment of trauma‐induced HO, as it is thought to result from the misguided differentiation of progenitor cells towards the bone instead of the tendon.^25^, ^32^

Different stages of HO involve various signal transduction pathways.^2^, ^25^ The mTORC1 pathway, an evolutionarily conserved mechanism controlling cell growth, can control mammalian skeletal growth by stimulating protein synthesis.^34^ Fu et al. recently revealed the activation of mTORC1 in the early stage of trauma‐induced HO.^35^ Building upon our previous investigation, the HO formation can be influenced by pharmaceutical interventions via the mTORC1 pathway in TDSCs.^7^ Consequently, it is suggested that MA can potentially intervene at the early stage of HO formation by modulating the mTORC1 in TDSCs, thereby preventing the progression of HO. This aligns with the imperative for early drug intervention in HO formation, emphasizing the avoidance of surgical procedures. Therefore, MA intervention was administrated 1 week after trauma‐induced Achilles tendon HO.^36^ The findings demonstrated that MA demonstrated an inhibitory effect on the elevated expression of p‐S6 in vivo and in vitro, indicating that suppressing the mTORC1 pathway in TDSCs might be a feasible strategy for alleviating HO. The mTORC1 complex consists of mammalian TOR (mTOR), regulatory‐associated protein of mTOR (Raptor), 40 kDa Pro‐rich AKT substrate (PRAS40), DEP domain‐containing mTOR‐interacting protein (Deptor) and mammalian lethal with SEC13 protein 8 (mLST8), and the complex is regulated upstream by Rheb (RAS homologue enriched in brain).^18^ The specific targets of MA inhibiting HO formation via regulating the mTORC1 pathway were investigated. HEK293T cells are a popular derivative of the original HEK293 parental cell line which enables them to produce recombinant proteins in plasmid vectors containing the SV40 promoter.^19^ Therefore, HEK293T cells were selected in this study for mechanistic investigation to transfect target proteins via plasmid vectors. Subsequently, the identified mechanism was further confirmed in the primary resident progenitor cells of TDSCs. The investigation demonstrated that MA inhibited the mTORC1 signalling pathway by promoting the degradation of Rheb protein and intercepting interaction between Rheb with mTOR.

Eukaryotes have evolved two major protein degradation systems, one is the ubiquitin‐proteasome system and the other one is the autophagy‐lysosome pathway, to maintain intracellular protein homeostasis.^37^ MA reduced Rheb protein expression without altering its transcriptional level, implying that MA induces Rheb protein degradation. Additionally, MA failed to expedite Rheb protein degradation in the presence of MG132 or MLN7243, indicating that MA facilitates Rheb protein degradation through the ubiquitin‐proteasome system. This aligns with previous reports that ubiquitination of Rheb negatively regulates mTORC1 activation.^20^ Protein ubiquitination involves the covalent linking of ubiquitin to substrate proteins, leading to the formation of 7 homotypic polyubiquitination, because ubiquitin contains 7 lysine residues suitable for ubiquitination, namely K6, K11, K27, K29, K33, K48 and K63.^38^, ^39^ Our findings indicate that MA selectively enhances the K48‐linked ubiquitination of Rheb, the most prevalent linkage in cells and recognized as the primary signal for proteasome‐mediated degradation.^40^ Ubiquitination is a reversible post‐translational modification orchestrated by an enzymatic cascade involving ubiquitin‐activating enzymes (E1), ubiquitin‐conjugating enzymes (E2) and ubiquitin ligases (E3), with reversal potential by deubiquitinases.^20^ Previous reports have identified RNF152 as a direct E3 ubiquitin ligase responsible for mediating Rheb ubiquitination, while USP4 acts as a deubiquitinase involved in Rheb deubiquitination, thereby bidirectionally regulating mTORC1 signalling pathway and influencing cell function.^20^ In our study, MA facilitates the K48‐linked ubiquitination of Rheb by modulating the deubiquitinase USP4. This regulation enhances the proteasomal degradation of Rheb protein and finally suppresses the mTORC1 signalling pathway. Consequently, MA facilitates the K48‐linked ubiquitin‐proteasomal degradation of Rheb through USP4 regulation, resulting in the inhibition of the mTORC1 signalling pathway and osteogenic differentiation of TDSCs, thereby serving as a potential treatment for trauma‐induced HO of Achilles tendon.

Although this study showed that MA could attenuate the trauma‐induced HO formation in Achilles tendon, it is constrained by a small sample size and experiments conducted in a small animal model. Expanding the research to include large animal models and large sample sizes would enhance the robustness of the conclusions. Additionally, the scope of this study is limited to the trauma‐induced HO model, and further exploration in relevant disease models is necessary to ascertain whether exhibits similar therapeutic effects in other types of HO.

## 4 MATERIALS AND METHODS

### 4.1 Reagents and Antibodies

The main reagents and antibodies used in this study are shown in Tables S1 and S2, respectively.

### 4.2 Animals and treatment

12‐week‐old male Sprague–Dawley rats were purchased from Guangdong Provincial Animal Center and then randomly divided into 4 groups (*n* = 6) (Sham, HO, HO+MA and Sham+MA group). The rat trauma‐induced HO model was established by the commonly used percutaneous Achilles tendon puncture method reported previously.^27^ Briefly, a 27‐gauge needle was percutaneously punctured laterally into the body of the right Achilles tendon after the rats were anaesthetised, and this procedure was repeated five times in the middle of the Achilles tendon body for each rat. For the Sham and Sham+MA groups, the needle was only passed through the skin without touching the Achilles tendon. After 1 week, rats were administered according to group‐dependent local injection of 80 μM (MA/saline) 100 μL (HO+MA, Sham+MA group) or 100 μL saline (Sham, HO group). Ten weeks after operation, rats were euthanized and the right hindlimb samples were harvested for the following studies. In this study, the preliminary experiment (*n* = 24) was used for μCT and histological assessments to evaluate the effect of MA in treating trauma‐induced HO, and the subsequent repeated experiment (*n* = 24) was used for qPCR and WB analyses to evaluate the expression of osteogenic differentiation‐related genes or proteins influenced by MA.

### 4.3 μCT analysis

The acquired right hindlimb samples were used for a μCT scan (μCT 80, Scanco Medical, Bruttisellen, Zurich, Switzerland) with parameter settings (60 kV, 150 μA, slice thickness 20 μm). The mimics software was used to perform 3D reconstruction and the Achilles tendon region was defined as the region of interest (ROI) in this study for bone volume analysis to quantify HO formation.

### 4.4 Histological evaluation

Harvested Achilles tendon specimens were fixed in 4% paraformaldehyde for 24 h and decalcified for 7 days using 10% ethylenediaminetetraacetic acid (pH 7.0). The specimens were then paraffin‐embedded and sectioned with a paraffin microtome (RM2125 RTS, Leica) to obtain 4 μm thick pathological sections. Haematoxylin and eosin (HE) staining and immunofluorescence staining were performed according to protocol and guidelines. Histological images were acquired by a scanning tissue microscope (Olympus BX51) and Image analysis software (Media Cybernetics Inc) was used to track and quantify HO areas based on the stained images.

### 4.5 Cell culture and cell viability assay

6‐week‐old male rats were utilized for the extraction of TDSCs. The extraction and culture method followed our previous study^7^, ^33^ and passage three cells were used for the present investigation. Cell Counting Kit‐8 (CCK‐8; Keygen Biotech) was used in this study to assess the effect of MA on cell proliferation.^7^, ^33^ TDSCs were seeded in 96‐well plates at a density of 1.0 × 10^4^ cells/well and various doses of MA (0, 20, 40 and 80 μM) were added after cell adhesion for treatment 24 h. The cell viability/proliferation assay of CCK‐8 reagent (10 μL/well) was added and cultured for 4 h. Absorbance was measured at 450 nm as suggested by the manufacturer.

### 4.6 Cell treatment

The cells were seeded in 6‐well plates at 2 × 10^6^ cells/well for 24 h, and then cells were treated with different doses of MA and IL‐1β (10 ng/mL; R&D Systems) for 24 h to mimic the microenvironment of trauma‐induced HO, and the cellular supernatant, RNA, and protein were collected for experiments.

### 4.7 Quantitative reverse transcription PCR (qRT‐PCR) assay

The experimental method was according to our previous study.^30^ Briefly, total RNA was extracted from Achilles tendon tissue or cells using TRIzol reagent (Thermo Fisher Scientific, Inc.), isolated using chloroform and isopropanol and then reverse transcribed into cDNA using the PrimeScript™ RT Kit (Cat. No. RR037A; Takara Bio, Inc.). qRT‐PCR was performed using an ABI Q6 Analyser with SYBR GreenER qRT‐PCR SuperMix Universal and Specific Primers. Expression levels were normalized to those of GAPDH housekeeping gene, and the data were analysed using the ΔΔ‐Ct method.^15^ The primers used in this study are listed in Table S3.

### 4.8 ALP staining and Alizarin red staining

TDSCs (1.0 × 10^5^/well) were cultured in six‐well plates and treated with IL‐1β 10 ng/mL alone or co‐treated with MA (20, 40 or 80 μM) in the presence of osteogenic medium (DMEM medium supplemented with 50 μM ascorbic acid, 0.1 μM dexamethasone and 10 mM β‐glycerol phosphate). After 7 and 14 days, ALP and Alizarin Red staining were performed, respectively. The experimental methods were based on our previous study.^7^, ^33^ After staining, a visual evaluation was performed with an Olympus BX51 microscope (Olympus Corporation).

### 4.9 Protein degradation inhibition and transfection experiments

The experimental procedures were based on our previous study.^30^ Regarding the protein degradation inhibition test, the autophagy inhibitor 3‐MA (10 mM) or proteasome inhibitor MG132 (10 μM) was added to the culture plates to inhibit the autophagy‐lysosome pathway or the ubiquitin‐proteasome system, respectively. Transient expression was achieved by cloning the plasmid into the pcDNA3.1 vector. HEK293T cells were transfected using Lipofectamine 2000 according to the protocol. Chemically synthesized sized 21 nucleotide siRNA duplexes were transfected using Lipofectamine RNAiMAX (TranSheepBio, Shanghai, China) according to the manufacturer's instructions (Table S4).

### 4.10 Western Blotting

Protein samples of Achilles tendon tissue or cells were prepared with lysates (Tris–HCl, pH 7.5, 1 M; EDTA 0.5 M; 10% SDS; NP‐40; sodium deoxycholate; CHAPS Triton X‐100) for western blotting analysis. The experimental method was according to our previous study.^30^ Briefly, according to the protocol, sample preparation, electrophoresis, PVDF membrane for transfer reaction, seal with 5% skim milk and incubation of primary antibody and secondary antibody were performed in sequence. Finally, chemiluminescence was detected using an enhanced chemiluminescence system (Cell Signalling Technology Inc).

### 4.11 Immunoprecipitation experiment

The IP experimental method is based on our previous experiments.^30^ Briefly, cell lysates were incubated with primary antibodies (4°C, overnight) followed by protein G‐agarose beads (4 h). The protein G beads was used to pull down the target proteins, which was then detected by WB method.

### 4.12 Proteasome activity assay

Upon IL‐1β (10 ng/mL) induction of TDSCs, protein samples were harvested for analysis. A total of 2 μg of protein samples was combined with 100 μM succinyl‐Leu‐Leu‐Val‐Tyr‐7‐amido‐4‐methylcoumarin (Suc‐LLVY‐AMC) in a proteasome activity buffer (composed of 50 mM Tris–HCl at pH 7.5, 40 mM KCl, 5 mM MgCl_2_, 1 mM DTT and 2 mM ATP) to achieve a final volume of 100 μL. The liberation of fluorescent‐free AMC was assessed with excitation at 380 nm and emission at 460 nm. Following a one‐hour incubation at 37°C in the absence of light, fluorescence was quantified using a spectrophotometer.^41^

### 4.13 Statistical

Data for each group was analysed using one‐way ANOVA with Tukey's post hoc method and the differences between the two groups were assessed through an unpaired *t*‐test with GraphPad Prism software version 8.0.1 (244) Software (Inc., San Diego, USA). The final results are expressed as mean ± SD. *p* < 0.05 was considered statistically significant.

## 5 CONCLUSIONS

This study demonstrates that MA can suppress the osteogenic differentiation of the resident progenitor cells of TDSCs in vitro and HO formation in vivo by inhibiting the mTORC1 signalling pathway, thereby inhibiting the progression of trauma‐induced HO of Achilles tendon in rats. Mechanistically, MA facilitates the degradation of Rheb protein through the K48 ubiquitination‐proteasome pathway by regulating deubiquitinase USP4 and also intercepts interaction between Rheb and mTORC1 complex, resulting in the inhibition of the mTORC1 signalling pathway and thereby the inhibition of the osteogenic differentiation of TDSCs. In summary, these findings suggest a potential for MA to attenuate trauma‐induced HO in the Achilles tendon of rats. Moreover, the present findings support the notion that targeting the mTORC1 pathway in TDSCs could be a viable approach for the treatment of trauma‐induced HO formation.

## AUTHOR CONTRIBUTIONS

**Huaji Jiang:** Conceptualization (equal); data curation (equal); formal analysis (equal); investigation (equal); methodology (equal); project administration (equal); validation (equal); writing – review and editing (equal). **Yan Ding:** Conceptualization (equal); investigation (equal); methodology (equal); writing – review and editing (equal). **Xuemei Lin:** Formal analysis (equal); methodology (equal); writing – review and editing (equal). **Qinyu Tian:** Software (equal); writing – review and editing (equal). **Yakui Liu:** Software (equal); writing – review and editing (equal). **Hebei He:** Investigation (equal). **Yongfu Wu:** Validation (equal); visualization (equal); writing – review and editing (equal). **Xinggui Tian:** Data curation (equal); writing – original draft (equal); writing – review and editing (equal). **Stefan Zwingenberger:** Formal analysis (equal); project administration (equal).

## FUNDING INFORMATION

This work was supported by a grant from China Postdoctoral Science Foundation (2022 M721504) and Guangdong Medical Research Foundation (A2022123).

## CONFLICT OF INTEREST STATEMENT

The authors declare no conflict of interest.

## INFORMED CONSENT STATEMENT

Not applicable.

## Supporting information


Figure S1.

**Data S1.**.

## Data Availability

The data presented in this study are available on request from the corresponding author.
