# Supplementary figures and images for: Malvidin attenuates trauma‐induced heterotopic ossification of tendon in rats by targeting Rheb for degradation via the ubiquitin‐proteasome pathway
Source: J Cell Mol Med. 2024 Apr 30;28(9):e18349. doi: 10.1111/jcmm.18349 (PMC11058603; doi:10.1111/jcmm.18349)

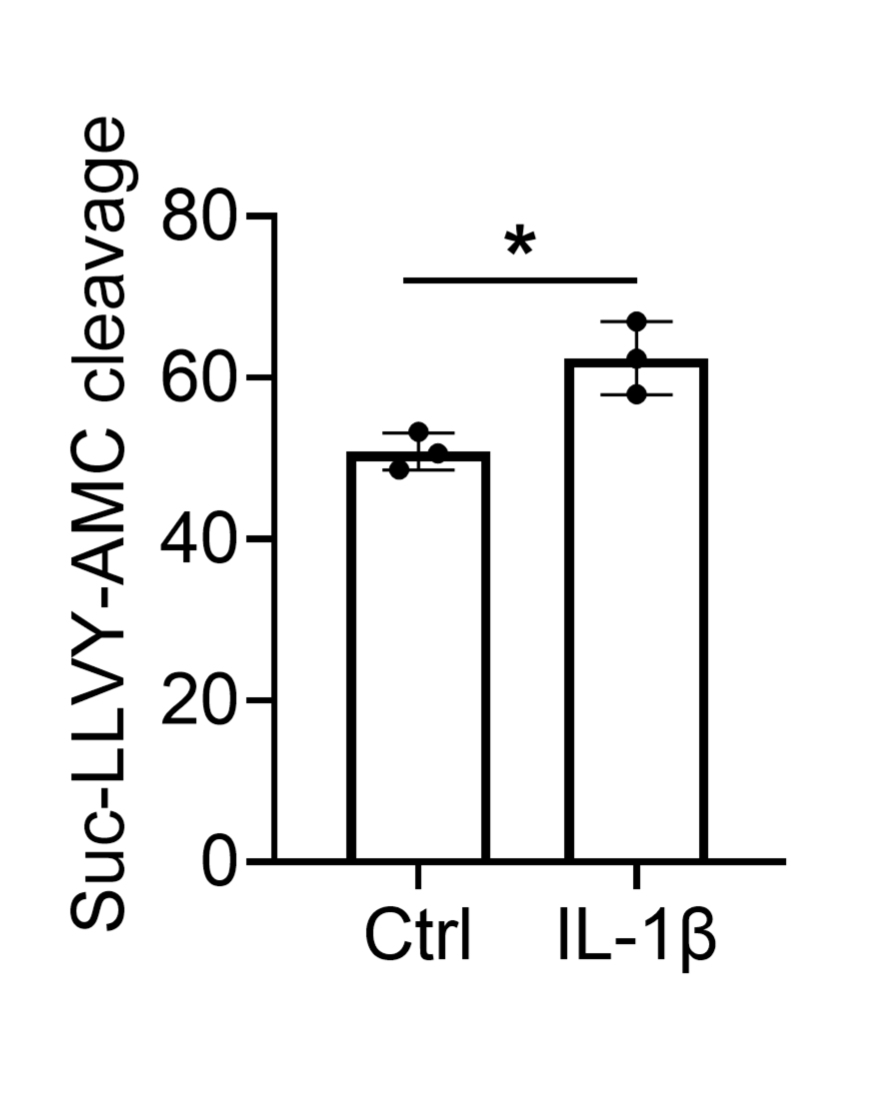

Supplement: Supplementary file 1 — Figure S1. [file JCMM-28-e18349-s002.tif]
