# Supplementary material for: Malvidin attenuates trauma‐induced heterotopic ossification of tendon in rats by targeting Rheb for degradation via the ubiquitin‐proteasome pathway
Source: J Cell Mol Med. 2024 Apr 30;28(9):e18349. doi: 10.1111/jcmm.18349 (PMC11058603; doi:10.1111/jcmm.18349)
Supplement: Supplementary file 2 — Data S1.. [file JCMM-28-e18349-s001.docx]

**Table S1** shows the reagents involved in this study.

| Reagents | Source | Identifier |
| --- | --- | --- |
| Malvidin | Sigma-Aldrich | 643-84-5 |
| Recombinant IL-1β | Beyotime | P6245 |
| MG-132 | Sigma-Aldrich | C-2211 |
| Chloroquine | Glpbio | 1954/5/7 |
| Cycloheximide | Sigma-Aldrich | C7698 |
| 3-methyladenine | Sigma-Aldrich | 5142-23-4 |
| MLN7243 | Sigma-Aldrich | HY-100487 |
| DMSO | Sigma-Aldrich | D2650 |
| Paraformaldehyde | Sigma-Aldrich | P6148 |
| ALP staining buffer | Beyotime Biotechnology | C3206 |
| Alizarin Red staining assay | Beyotime Biotechnology | C0148S |
| Lipofectamine 2000 | ThermoFisher | 11668019 |
| Protein G beads | GenScript | L00209 |
| Type I collagenase | Wako | 031-17601 |
| Hematoxylin and eosin dyes | Beyotime | C0105S |
| Masson's trichrome kit | Solarbio | G1340 |
| TRIZOL | Beyotime Biotechnology | R0016 |
| Fetal bovine serum | Sigma-Aldrich | 12103C |
| Amphotericin B | Gibco | 15290026 |
| Trypsinization | Gibco | R001100 |
| Cell Counting Kit-8 | Beyotime Biotechnology | C0037 |
| Penicillin/streptomycin | Gibco | 15140122 |
| Lipofectamine RNAiMAX | Invitrogen | 13778150 |
| Enhanced chemiluminescence kit | Cell Signaling Technology | 12630 |

**Table S2** shows the antibodies involved in this study.

| Antibodies | Source | Identifier |
| --- | --- | --- |
| RUNX2 | Abclonal | A2851 |
| OCN | Abclonal | A18241 |
| p-S6 | Cell Signaling Technology | 4858 |
| S6 | Cell Signaling Technology | 2317 |
| Rheb | Santa Cruz Biotechnology | sc-271509 |
| Raptor | Santa Cruz Biotechnology | sc-81537 |
| Anti-Actin | Abclonal | AC004 |
| Anti-Flag agarose gels | Sigma-Aldrich | A2220 |
| Horseradish peroxidase (HRP)-anti-Flag (M2) | Sigma-Aldrich | F1804 |
| Anti-HA | Roche Applied Science | 12994 |
| Anti-Ub | Santa Cruz Biotechnology | sc-8017 |
| Anti-K48 Ub | Abclonal | A3606 |
| Goat anti-rabbit IgG (H&L) | Santa Cruz Biotechnology | sc-2004 |
| Goat anti-mouse IgG (H&L) | Santa Cruz Biotechnology | sc-2005 |
| Goat anti-rabbit IgG (H&L) Alexa Fluor 488 | Invitrogen | A11029 |
| Goat anti-rabbit IgG (H&L) Alexa Fluor 594 | Immunoway | RS23420 |

**Table S3**: Primers for qRT-PCR used in this study.

| Gene | Sequences | Species |
| --- | --- | --- |
| Runx2 | Forward: GTCGTCAGACCGAGAAGTGG  Reverse: TCAAGTTCGAGGAAGCCGTG | Rat |
| Osx | Forward: GCTTTTCTGTGGCAAGAGGTTC  Reverse: CTGATGTTTGCTCAAGTGGTCG | Rat |
| Ocn | Forward: CGCGTAAACGCCCTTTTGAT  Reverse: AGTCTTGCAGCACCCGTAAA | Rat |
| Alp | Forward: CGTCTCCATGGTGGATTATGC  Reverse: TGGCAAAGACCGCCACAT | Rat |
| Rheb | Forward: GACCTGCATATGGAAAGGGTGA  Reverse: CAGGCGCGTCACATCA | Rat |
| Gapdh | Forward: GGCATTGCTCTCAATGACAA  Reverse: TGTGAGGGAGATGCTCAGTC | Rat |

**Table S4** shows the sequence of all target siRNAs.

| Gene | Sequences |
| --- | --- |
| RNF152 siRNA | 5′-GCUGGAAUGUCAGAUCUGU-3′  3′-GCUUCACUGUGAUAUCCUG-5’ |
| USP4 siRNA | 5′-GCAAAUGGUGAUAGCACUATT-3′  3′-UAGUGCUAUCACCAUUUGCTT-5’ |


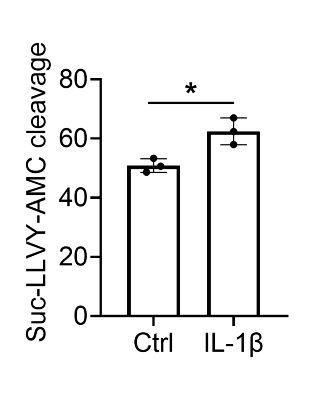


**Figure S1.** The effects of IL-1β alone on the proteasome system of tendon-derived stem cells (TDSCs). Data are expressed as the means ± SD. * p < 0.05.
